# CDK Inhibition Reverses Acquired 5-Fluorouracil Resistance in Hepatocellular Carcinoma Cells

**DOI:** 10.1155/2022/6907057

**Published:** 2022-03-11

**Authors:** Yiyi Pu, Dongmei Yan, Linglan Tu, Liyan Cheng, Jie Yu, Zhuduo Li, Xiaoliang Zheng, Xinbao Wang

**Affiliations:** ^1^Department of the Hepato-Biliary-Pancreatic Surgery, The Cancer Hospital of the University of Chinese Academy of Sciences (Zhejiang Cancer Hospital), Hangzhou, Zhejiang, China; ^2^School of Bioengineering, Hangzhou Medical College, Hangzhou, Zhejiang, China

## Abstract

**Background:**

5-Fluorouracil (5-FU) has been widely applied in treating cancers. However, its usage is largely limited in hepatocellular carcinoma (HCC), due to acquired resistance. Here, we aim to identify target proteins and investigate their roles in 5-FU sensitivity of HCC cells.

**Methods:**

Mass spectrometry (MS) proteomics was performed on 5-FU-resistant cell line (BEL7402/5-FU) and its parental cell line (BEL7402) with 5-FU treatment. In order to identify potential targets, we compared the proteomics between two cell line groups and used bioinformatics tools to select hub proteins from all differentially expressed proteins.

**Results:**

We finally focused on a group of cell cycle-related kinases (CDKs). By CCK8 assay, we confirmed that the CDK inhibitor significantly decreased the IC_50_ of 5-FU-resistant cells.

**Conclusions:**

Our study verified that CDK inhibition can reverse 5-FU resistance of HCC cells.

## 1. Introduction

Liver cancer is the sixth most frequent cancer and the fourth leading cause of cancer death worldwide. For males, liver cancer has the second highest mortality rate [1]. Hepatocellular carcinoma (HCC), as the primary subtype, comprises 75%-85% [1] of all liver cancer cases. Chemotherapy is a traditional way of treating cancers. However, its application is limited in HCC treatment, mainly due to chemoresistance [2]. 5-Fluorouracil (5-FU) is one widely used chemotherapy drug which implements its anticancer function by inhibiting thymidylate synthase (TS) and incorporating its metabolites into nucleic acid molecules [3]. However, with severe resistance, 5-FU's application is largely limited in HCC. To overcome this problem, efforts have been made to explore the mechanism of 5-FU resistance in HCC and several involved genes were identified, such as SIX1 [4], RBFOX3 [5], and BCL6B [6]. Noncoding RNAs also play a role, such as microRNA-122 [7], lncRNA HULC [8], and lncRNA KRAL [9]. However, the mechanism of 5-FU resistance is still far from clear, and it remains a great challenge to reverse such resistance.

MS proteomics has been extensively applied in various aspects of cancer research, including mechanism investigation, molecular subtype definition, and biomarker identification [10]. Based on MS proteomics, there were already several studies about 5-FU resistance in HCC [11–13]. These studies quantitatively compared the proteomes of HCC cells with 5-FU-resistant HCC cells and identified differential expressed proteins. Different from previous studies, we aim to investigate the proteomes with 5-FU treatment. Thus, we conducted a comparative proteomics study between 5-FU-resistant HCC cell line (BEL7402/5-FU) and its parental cell line (BEL7402), both treated with 5-FU. We found 129 differentially expressed proteins, identified CDK1 (cyclin dependent kinase 1) as a hub protein by bioinformatics tools, and validated that CDK inhibition can reverse the 5-FU resistance of BEL7402/5-FU cells.

## 2. Materials and Methods

### 2.1. Cell Lines and Cultures

Human hepatocellular carcinoma cells (BEL7402) were supplied by Chinese Academy of Sciences (Shanghai, China). The 5-FU-resistant strain (BEL7402/5-FU) was successfully induced by increasing 5-FU concentration from 0.5 *μ*mol/L to 150 *μ*mol/L in the culture medium. BEL7402 cells and BEL7402/5-FU cells were grown in RPMI 1640 medium supplemented with 10% fetal bovine serum at 37°C in a saturated humidified incubator containing 5% CO_2_ [14]. All cell lines were verified by short tandem repeat (STR) profiling. The concentration of 5-FU treatment was set to be the IC_50_ of BEL7402 cells (3 *μ*M). Two groups were established from BEL7402 cells and BEL7402/5-FU cells under 5-FU treatment with two replicates per group.

### 2.2. Protein Extraction and Digestion

BEL7402 or BEL7402/5-FU cells (>5∗10^6^) with 5-FU treatment were sonicated three times on ice using a high intensity ultrasonic processor (Scientz) in lysis buffer with 8 M urea and 1% Protease Inhibitor Cocktail. For each sample, the protein was collected by centrifugation at 12,000g at 4°C for 10 min. 450 *μ*g protein solution was digested with 5 mM dithiothreitol for 30 min at 56°C and alkylated with 11 mM iodoacetamide for 15 min at room temperature in darkness and then diluted by 100 mM TEAB. For protein digestion, trypsin was added at 1 : 50 trypsin-to-protein mass ratio overnight and 1 : 100 ratio for 4 h.

### 2.3. Peptide Labeling, Fractionation, and LC-MS/MS Analysis

According to the manufacturer's protocol for TMT kit, the tryptic peptides were desalted by Strata X C18 SPE column (Phenomenex) and vacuum-dried, followed by reconstituted in 0.5 M TEAB. Use Thermo Betasil C18 column (5 *μ*m particles, 10 mm in inner diameter, 250 mm in length) to fractionate the tryptic peptides into parts by high pH reversed-phase HPLC, then dissolve them in 0.1% formic acid (solvent A), and load them directly into a reversed-phase analytical column (15 cm in length, 75 *μ*m in inner diameter). The gradient includes an increase from 6% to 23% solvent B (0.1% formic acid in 98% acetonitrile) in 26 min, an increase from 23% to 35% in 8 min, an increase to 80% in 3 min, and then holding at 80% in the last 3 min. All of the above were performed on the EASY-nLC 1000 UPLC system at a constant flow rate of 400 nL/min. The peptides are passed through an NSI source and then subjected to tandem mass spectrometry (MS/MS) in Q Exactive™ Plus (Thermo) and coupled to UPLC online.

### 2.4. Database Searching and Data Analysis

The resulting MS/MS data were processed using Maxquant search engine (v.1.5.2.8). Tandem mass spectra were searched against human uniprot database concatenated with reverse decoy database. Calculate the quantitative value of peptides in each sample based on the ratio of the labeled reporter ion intensity in the MS/MS spectrum of the original dataset. The protein content in each sample is calculated as the median of the unique peptides of the specific protein. The quantitative ratio of protein between the two samples is considered the protein expression ratio. In order to calculate the *p* value of the differentially expressed protein between samples, the log 2 transformation was performed on the unique peptide quantitative value of the protein in the two samples to make the data be normally distributed, and then, the two-tailed *t*-test was used for the two samples. The Benjamini-Hochberg method was used to adjust *p* values. Proteins with false discovery rate (FDR) < 0.05 and expression ratio > 1.5 (or <1/1.5) were regarded as differentially expressed.

### 2.5. Functional and Pathway Enrichment Analysis

Gene ontology (GO) analyses and Kyoto Encyclopedia of Genes and Genomes (KEGG) pathway enrichment analyses for identified proteins were carried out through Search Tool for Retrieval of Interacting Genes/Proteins (STRING, version 11.0) [15]. Enriched GO terms and pathways were considered significant with FDR lower than 0.05.

### 2.6. Protein-Protein Interaction (PPI) Network

A PPI network was built using STRING database to identify key proteins. In order for a credible network, interaction score cutoff was set as 0.7 and only four reliable active interaction sources (Experiments, Databases, Co-expression, and Textmining) were selected. The “NetworkAnalyzer” tool in Cytoscape (3.7.1) [16] was then used to calculate degree centrality for all nodes in the PPI network.

### 2.7. Western Blotting

The total proteins of each cell type were extracted with RIPA lysis buffer. The protein concentrations were measured using the Bradford kit (Beyotime Biotechnology, Nantong, China). Aliquots of 30 *μ*g total protein were boiled for 5 min in loading buffer, then separated by 12.5% SDS-PAGE and transferred onto nitrocellulose membranes. The membranes were blocked with blocking buffer (5% skimmed milk in TBST) and incubated with primary antibodies (Cell Signaling Technology, Danvers, MA, USA) (overnight at 4°C) followed by secondary antibodies (Cell Signaling Technology, Danvers, MA, USA). Finally, BeyoECL Plus was used for the protein bands developing.

### 2.8. Cell Counting Kit-8 (CCK-8) Assay

Cell viability was measured by CCK-8 assay (Yeasen Biotech Co., Ltd., Shanghai, China). Cells treated with or without CDK inhibitor Dinaciclib (Topscience Biotechnology, Shanghai, China) were plated in 96-well plates at 2 × 10^3^ cells per well. After 24 h incubation at 37°C and 5% CO_2_, 5-FU was added at different concentrations. After 72 h incubation, the cells were incubated with 10 *μ*L/well CCK8 solution for 2 h. Finally, the light absorbance was measured by Microplate Reader (Synergy 2, BioTek Instruments, Inc., USA) at 450 nm. Based on the inhibition rate, the IC_50_ values were calculated by GraphPad Prism (5.01).

## 3. Results

### 3.1. Identification of Differentially Expressed Proteins

**Figure 1:**
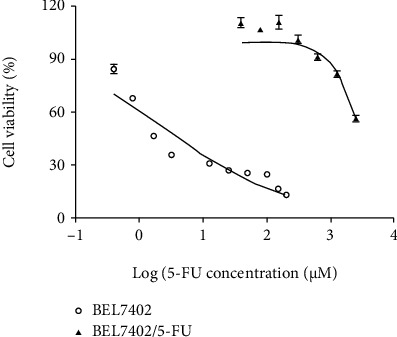
Quantity-effect relationship graph of BEL7402 cells and BEL7402/5-FU cells towards 5-FU. The *X* axis and *Y* axis show the log transformation of 5-FU concentration and the cell viability, respectively. Error bars show the standard error (SE).

**Figure 2:**
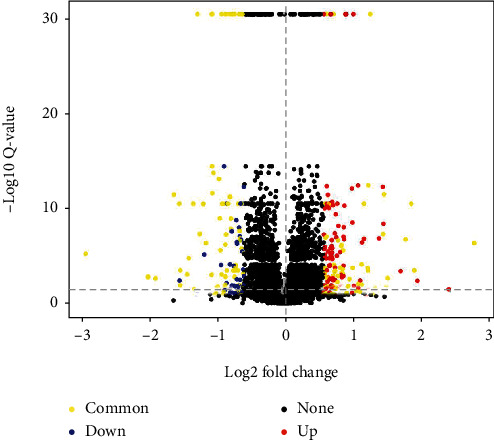
Volcano plots of proteins in MS proteomics experiments. Red and blue dots show upregulated and downregulated proteins in 5-FU-resistant cells in one parallel experiment. Yellow dots show 129 commonly differentially expressed proteins.

**Figure 3:**
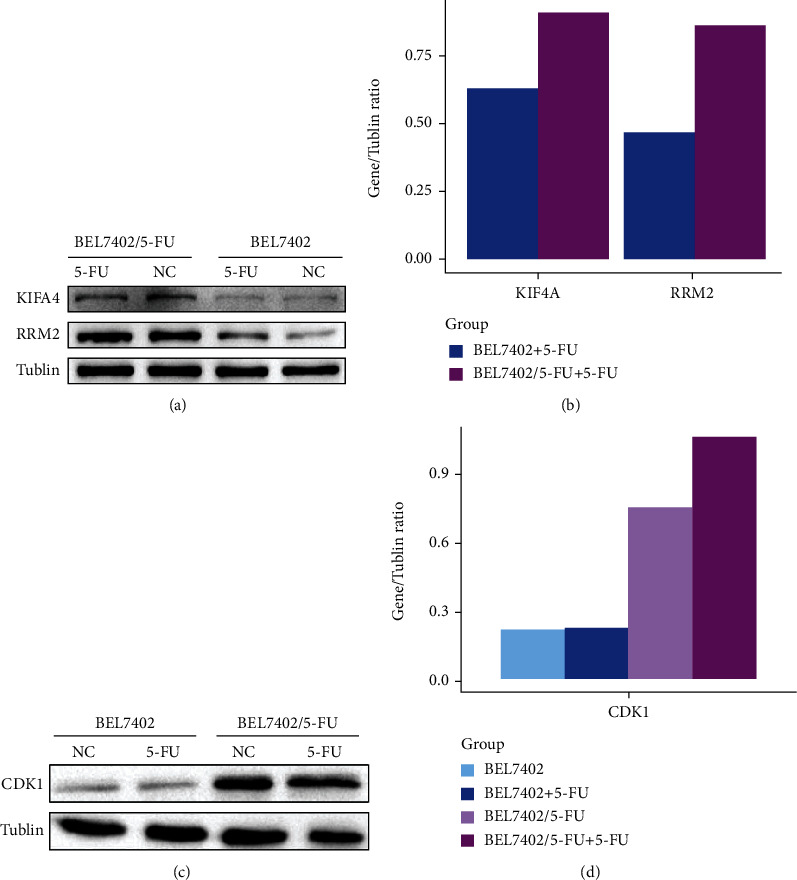
Western blot of BEL7402 cells and BEL7402/5-FU cells without and with 3 *μ*mol/L 5-FU treatment. (a) Western blot results of KIF4A, RRM2, and tubulin. (b) Gray values of KIF4A, RRM2 western blot results. (c) Western blot results of CDK1 and tubulin. (d) Gray values of CDK1 western blot result.

At first, we compared the sensitivity of BEL7402 cells and BEL7402/5-FU cells towards 5-FU (Figure 1). The 5-FU IC_50_ for BEL7402 cells and BEL7402/5-FU cells were 3.00 ± 0.98 *μ*M and 2758.50 ± 167.58 *μ*M, respectively. Based on a nearly 920 folds higher IC_50_ than BEL7402 cells, BEL7402/5-FU cells were confirmed to have obtained strong 5-FU resistance. To identify 5-FU resistance-related proteins and pathways activated under drug condition, we performed comparative quantitative proteomics analyses on 6425 unique proteins between BEL7402/5-FU cells and BEL7402 cells with 5-FU treatment (3 *μ*M). In order to get reliable results, we repeated the whole process twice. Differentially expressed proteins (fold change > 1.5, FDR < 0.05) identified from each experiment were compared, and 129 commonly differentially expressed proteins (yellow in Figure 2) were finally determined. We further selected 3 differentially expressed proteins (KIF4A, RRM2, and CDK1) from MS proteomics and validated their expression by western blotting (Figure 3). We found under 5-FU treatment (3 *μ*M) that the expression of 3 proteins was higher in 5-FU-resistant cells (BEL7402/5-FU) than sensitive cells (BEL7402) which agreed with the MS proteomics results (Table 1).

### 3.2. Functional and Pathway Enrichment Results of Differentially Expressed Proteins

**Figure 4:**
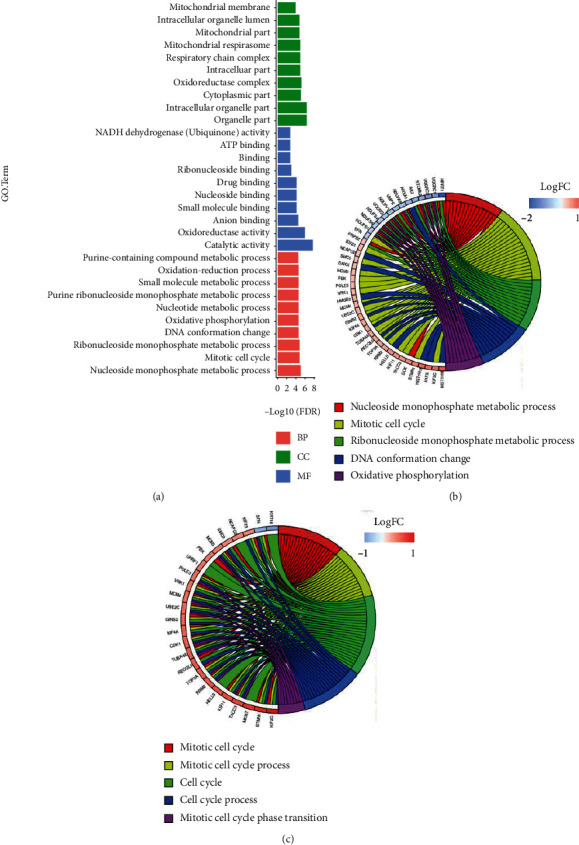
GO enrichment of 129 differentially expressed proteins. (a) Top 10 enriched GO terms of 129 proteins, with Biological Processes in red, Molecular Functions in blue, and Cellular Components in green. (b) The relation between top 5 enriched BP terms and proteins displayed by chord plot. (c) The relation between all cell cycle-relevant-enriched BP terms and proteins displayed by chord plot. Proteins are labeled along the left half circle on each plot, and the color of corresponding box shows the logFC value (red: upregulated genes in BEL7402/5-FU cells; blue: downregulated genes in BEL7402/5-FU cells).

Genes encoding all 129 proteins were included in GO analyses. There were a large number of enriched GO terms (FDR < 0.05), including 85 Biological Processes (BP), 44 Molecular Functions (MF), and 53 Cellular Components (CC) (top 10 terms of each category are shown in Figure 4(a)). Cell cycle relevant terms were among the top BP terms, such as “mitotic cell cycle,” “mitotic cell cycle process,” “cell cycle” and “cell division.” Interestingly, top BP terms were enriched by certain group of genes (Figure 4(b)). Cell cycle relevant terms were all enriched by upregulated genes (Figure 4(c)), while terms like “nucleoside monophosphate metabolic process,” “ribonucleoside monophosphate metabolic process,” and “oxidative phosphorylation” were mostly enriched by downregulated genes.

**Figure 5:**
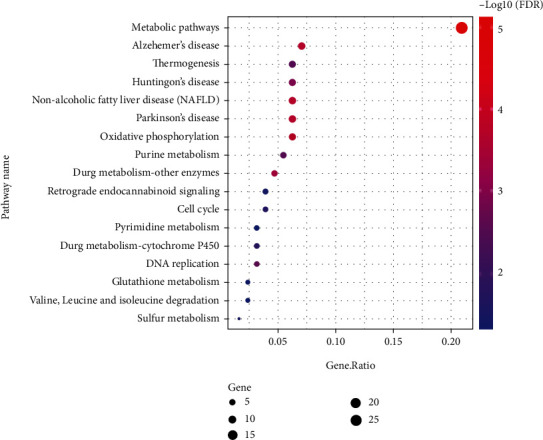
Enriched KEGG pathways for all 129 differentially expressed proteins.

There were 17 enriched KEGG pathways (Figure 5), including specific ones like “Oxidative phosphorylation,” “DNA replication,” “Purine metabolism,” “Cell cycle,” “Sulfur metabolism,” “Retrograde endocannabinoid signaling,” and “Valine, leucine and isoleucine degradation”.

### 3.3. CDK1 Was Identified as the Hub Protein by PPI Network

**Figure 6:**
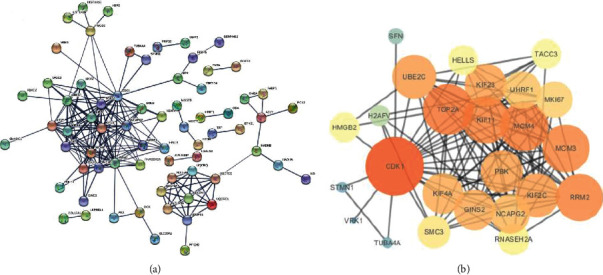
PPI network of all 129 differentially expressed proteins. (a) PPI network from STRING. (b) PPI network of CDK1-related proteins by Cytoscape, with orange color and large circle size representing high degree centrality.

PPI network based on 129 proteins was constructed by STRING (11.0) (Figure 6(a)) and analyzed by Cytoscape (3.7.1). According to the centrality of all nodes, hub proteins were defined as the ones with high degree centrality (the number of links upon nodes). With the highest degree centrality (links to 24 proteins out of total 69 proteins, Figure 6(b)), CDK1 was identified as the hub protein. Besides CDK1, several other cell cycle relevant proteins were also identified, such as MCM3, MCM4, SFN, and SMC3.

### 3.4. CDK Inhibition Increased 5-FU Sensitivity of BEL7402/5-FU Cells

Besides proteomics analysis, we confirmed the high expression of CDK1 in 5-FU-resistant cells by western blot (Figures 3(c) and 3(d)). Our results showed that 5-FU-resistant cells possessed higher CDK expression with or without 5-FU treatment.

**Figure 7:**
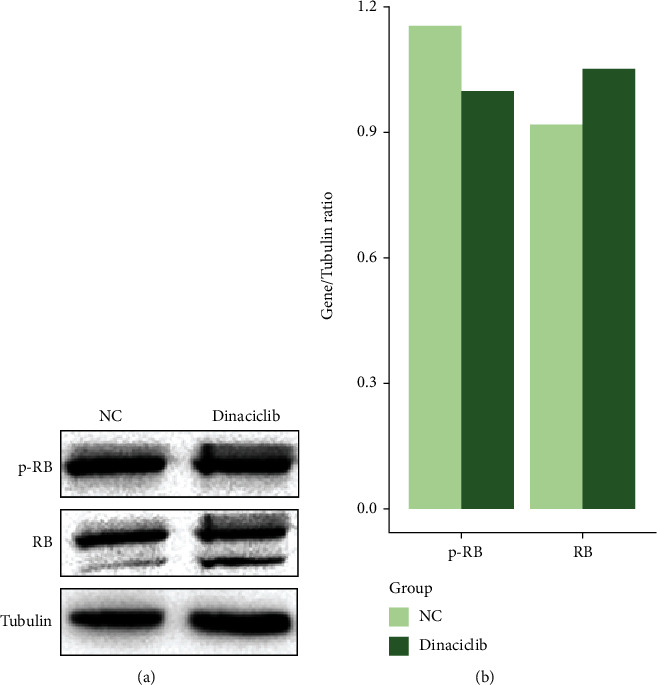
Western blot of BEL7402/5-FU cells with 5-FU treatment (NC) and 5-FU-Dinaciclib-combined treatment (Dinaciclib). p-RB: phosphorylated RB; RB: total RB. (a) Western blot results of p-RB and RB. (b) Gray values of western blot results.

**Figure 8:**
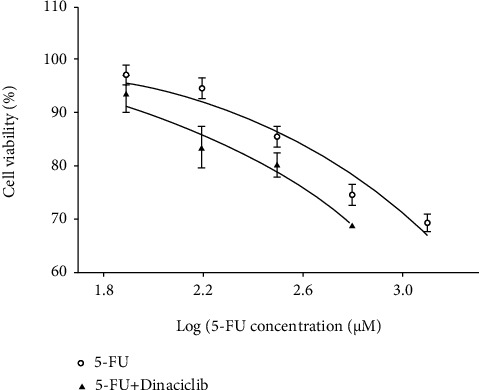
Quantity-effect relationship graph of BEL7402/5-FU cells with 5-FU treatment and 5-FU-Dinaciclib combined treatment. The *X* axis and *Y* axis show the log transformation of 5-FU concentration and the cell viability, respectively. Error bars show the SE.

The CDK inhibitor (Dinaciclib) was previously proved to largely decrease the activity of CDK1, CDK2, CDK5, and CDK9 *in vitro* [17]. In order to verify the efficacy of Dinaciclib, we detected the phosphorylation level of retinoblastoma-associated protein (RB) which is the downstream protein of CDKs [18]. Based on our western blot results, similar to Dinaciclib's original paper [17], Dinaciclib decreased RB's phosphorylation and in the meantime increased RB's total expression (Figure 7). By CCK8 assay, we confirmed that the addition of Dinaciclib (10 nmol/L) significantly reduced the IC_50_ of BEL7402/5-FU cells (5-FU: 2650.75 ± 242.48 *μ*M; 5-FU+Dinaciclib: 1941.25 ± 424.82 *μ*M; *p* = 0.032, Figure 8) towards 5-FU without significant cytotoxicity during Dinaciclib treatment alone.

## 4. Discussion

5-FU is a commonly used chemotherapy drug. However, in treating HCC, both 5-FU-based monotherapy and combination chemotherapy did not achieve high response rates [19–22]. Numerous studies have been conducted to explore the mechanism of 5-FU resistance and also try to reverse such resistance [4–9]. MS proteomics, an approach for broad detection, has been widely used in identifying cancer target molecules, including the following studies about 5-FU resistance mechanism in HCC cells. After comparing 5-FU-resistant HCC cell line with its parental cell line, Tong et al. identified 52 differentially expressed proteins and verified that ANXA3 correlates with 5-FU resistance [13]. Similarly, from 102 differentially expressed proteins, Tan et al. verified that downregulation of PRDX6 and PSMB7 can increase sensitivity towards 5-FU [12]. Conducting proteomic and phosphoproteomic approaches, Liu et al. identified 2326 differentially expressed proteins and 8614 differentially phosphorylated sites. Finally, they focused on GnRH signaling pathway and confirmed that the knockdown of PLC*β*3, PKC*δ,* and SRC could increase 5-FU sensitivity [11]. The above studies proved that comparing the proteomes of 5-FU-sensitive cells with resistant cells can identify effective target proteins. However, 5-FU itself as a stimulus may largely alter cell physiology. And by definition, the significant difference between sensitive and resistant cell type is their responses towards 5-FU. Thus, we performed the quantitative proteomics method on those two cell types under 5-FU treatment. Based on a threshold of 1.5-fold change, we identified 129 significantly differentially expressed proteins after comparing results from two replicates. Based on three primary reasons, we finally focused on CDK family. First, cell cycle was among the top BP terms in GO analysis. Second, cell cycle pathway was one of the enriched KEGG pathways. Last and the most importantly, CDK1 was identified to be the hub protein in PPI network.

Cyclin-dependent kinases (CDKs), together with cyclins and CDK inhibitors, play indispensable roles in cell cycle control and also in other processes such as transcription, DNA damage repair, proteolytic degradation, and epigenetic regulation [23]. Cell cycle deregulation is associated with resistance towards multiple drugs, including 5-FU [24]. The CDK inhibitor we used here was Dinaciclib which inhibits the activity of CDK1, CDK2, CDK5, and CDK9 [17]. Due to the potential of CDKs as the drug target, since 2006, Dinaciclib (SCH 727965) (https://clinicaltrials.gov/ct2/results?term=Dinaciclib) has entered 18 clinical trials for treating cancers like leukemia, breast cancer and pancreatic cancer, myeloma, and melanoma. Here, our results showed that Dinaciclib was also able to reverse the 5-FU resistance in HCC cells.

Previous studies gave inconsistent results about the correlation between CDK and 5-FU resistance. Consistent with our results, Takagi et al. found that the CDK inhibitor SU9516 upregulated the sensitivity of colorectal cancer cells to 5-FU [25]. Using another agent, Chen et al. found that a Chinese herbal (Hedyotis diffusa Willd) could enhance the antitumor effect of 5-FU towards HCC cells by downregulating CDK2 and E2F1 [26]. Contradictory results were also reported. By miR-381, Chen et al. sensitized renal cancer cells to 5-FU through WEE1 inhibition and CDK1 activation [27]. RB, as the downstream protein of CDKs [18], was confirmed to be partially dephosphorylated by Dinaciclib treatment (Figure 7). The hypophosphorylated RB could further bind to and downregulate transcription factor E2F1 [28]. Then the downregulation of E2F1 could cause the low expression of its target gene thymidylate synthase (TS) [29] which is known as the key enzyme in 5-FU's anticancer effect [30]. Similar stories have been told by other studies. For example, Takagi et al. found that the CDK inhibitor could significantly reduce TS expression [25]. Watanabe et al. also successfully enhanced 5-FU efficacy by RB-reactivating agents companied by TS downregulation [31]. In summary, the CDK-RB-E2F1-TS axis was likely to play a part in our scenario.

## 5. Conclusions

In conclusion, by comparative MS proteomics between 5-FU-sensitive and 5-FU-resistant cells with 5-FU treatment, we identified CDK1 as the hub protein and verified that CDK inhibition can reverse acquired 5-FU resistance in HCC cells.

## Figures and Tables

**Table 1:** MS proteomics result of validated proteins from two experiment replicates.

Protein	Re/Se ratio 1	FDR 1	Re/Se ratio 2	FDR 2
KIF4A	1.78	0.00018	4.94	5.04*e* − 09
RRM2	1.83	0.00079	4.21	6.92*e* − 07
CDK1	1.79	9.55*e* − 07	3.73	2.33e-15

Re/Se ratio: the ratio of protein amount between BEL7402/5-FU and BEL7402.

## Data Availability

The CCK8 assay results for Figure 1 and Figure 8 are provided in supplementary file 1..

## Supplementary Materials

Supplementary Materials Supplementary materials include CCK8 assay results for Figure 1 (BEL7402 vs. BEL7402/5-FU cells treated with 5-FU) and Figure 8 (BEL7402/5-FU cells treated with 5-FU vs. 5-FU-Dinaciclib).

## References

[1] Bray F., Ferlay J., Soerjomataram I., Siegel R. L., Torre L. A., Jemal A. (2018). Global cancer statistics 2018: GLOBOCAN estimates of incidence and mortality worldwide for 36 cancers in 185 countries. *CA: a Cancer Journal for Clinicians*.

[2] Lohitesh K., Chowdhury R., Mukherjee S. (2018). Resistance a major hindrance to chemotherapy in hepatocellular carcinoma: an insight. *Cancer Cell International*.

[3] Longley D. B., Harkin D. P., Johnston P. G. (2003). 5-Fluorouracil: mechanisms of action and clinical strategies. *Nature Reviews. Cancer*.

[4] Chen K., Wei H., Pan J. (2019). Six1 is negatively correlated with poor prognosis and reduces 5-fluorouracil sensitivity via attenuating the stemness of hepatocellular carcinoma cells. *European Journal of Pharmacology*.

[5] Liu T., Wu X., Li Y. (2018). RBFOX3 regulates the chemosensitivity of cancer cells to 5-fluorouracil via the PI3K/AKT, EMT and cytochrome-C/caspase pathways. *Cellular Physiology and Biochemistry*.

[6] Li X., Yu J., Brock M. V. (2015). Epigenetic silencing of BCL6B inactivates p53 signaling and causes human hepatocellular carcinoma cell resist to 5-FU. *Oncotarget*.

[7] Wang W., Liu W. B., Huang da B., Jia W., Ji C. S., Hu B. (2016). Targeting PCDH20 gene by microRNA-122 confers 5-FU resistance in hepatic carcinoma. *American Journal of Cancer Research*.

[8] Xiong H., Ni Z., He J. (2017). LncRNA HULC triggers autophagy via stabilizing Sirt1 and attenuates the chemosensitivity of HCC cells. *Oncogene*.

[9] Wu L., Pan C., Wei X. (2018). lncRNA KRAL reverses 5-fluorouracil resistance in hepatocellular carcinoma cells by acting as a ceRNA against miR-141. *Cell Communication and Signaling: CCS*.

[10] Timms J. F., Hale O. J., Cramer R. (2016). Advances in mass spectrometry-based cancer research and analysis: from cancer proteomics to clinical diagnostics. *Expert Review of Proteomics*.

[11] Liu Z., Wang Y., Yao Y., Fang Z., Miao Q. R., Ye M. (2019). Quantitative proteomic and phosphoproteomic studies reveal novel 5-fluorouracil resistant targets in hepatocellular carcinoma. *Journal of Proteomics*.

[12] Tan Y., Qin S., Hou X. (2014). Proteomic-based analysis for identification of proteins involved in 5-fluorouracil resistance in hepatocellular carcinoma. *Current Pharmaceutical Design*.

[13] Tong S. W., Yang Y. X., Hu H. D. (2012). Proteomic investigation of 5-fluorouracil resistance in a human hepatocellular carcinoma cell line. *Journal of Cellular Biochemistry*.

[14] Gu W., Fang F. F., Li B., Cheng B. B., Ling C. Q. (2012). Characterization and resistance mechanisms of a 5-fluorouracil- resistant hepatocellular carcinoma cell line. *Asian Pacific Journal of Cancer Prevention*.

[15] Szklarczyk D., Gable A. L., Lyon D. (2019). STRING v11: protein-protein association networks with increased coverage, supporting functional discovery in genome-wide experimental datasets. *Nucleic Acids Research*.

[16] Shannon P., Markiel A., Ozier O. (2003). Cytoscape: a software environment for integrated models of biomolecular interaction networks. *Genome Research*.

[17] Parry D., Guzi T., Shanahan F. (2010). Dinaciclib (SCH 727965), a novel and potent cyclin-dependent kinase inhibitor. *Molecular Cancer Therapeutics*.

[18] Giacinti C., Giordano A. (2006). RB and cell cycle progression. *Oncogene*.

[19] Tetef M., Doroshow J., Akman S. (1995). 5-Fluorouracil and high-dose calcium leucovorin for hepatocellular carcinoma: a phase II trial. *Cancer Investigation*.

[20] Porta C., Moroni M., Nastasi G., Arcangeli G. (2004). 5-Fluorouracil and <i>d, </i><i>l</i>-Leucovorin calcium are active to treat unresectable hepatocellular carcinoma patients: preliminary results of a phase II study. *Oncology*.

[21] Park S. H., Lee Y., Han S. H. (2006). Systemic chemotherapy with doxorubicin, cisplatin and capecitabine for metastatic hepatocellular carcinoma. *BMC Cancer*.

[22] Qin S., Bai Y., Lim H. Y. (2013). Randomized, multicenter, open-label study of oxaliplatin plus fluorouracil/leucovorin versus doxorubicin as palliative chemotherapy in patients with advanced hepatocellular carcinoma from Asia. *Journal of Clinical Oncology*.

[23] Lim S., Kaldis P. (2013). Cdks, cyclins and CKIs: roles beyond cell cycle regulation. *Development*.

[24] Shah M. A., Schwartz G. K. (2001). Cell cycle-mediated drug resistance: an emerging concept in cancer therapy. *Clinical Cancer Research*.

[25] Takagi K., Sowa Y., Cevik O. M., Nakanishi R., Sakai T. (2008). CDK inhibitor enhances the sensitivity to 5-fluorouracil in colorectal cancer cells. *International Journal of Oncology*.

[26] Chen X. Z., Cao Z. Y., Chen T. S. (2012). Water extract of Hedyotis Diffusa Willd suppresses proliferation of human HepG2 cells and potentiates the anticancer efficacy of low-dose 5-fluorouracil by inhibiting the CDK2-E2F1 pathway. *Oncology Reports*.

[27] Chen B., Duan L., Yin G., Tan J., Jiang X. (2013). miR-381, a novel intrinsic WEE1 inhibitor, sensitizes renal cancer cells to 5-FU by up-regulation of Cdc2 activities in 786-O. *Journal of Chemotherapy*.

[28] Brown V. D., Phillips R. A., Gallie B. L. (1999). Cumulative effect of phosphorylation of pRB on regulation of E2F activity. *Molecular and Cellular Biology*.

[29] Kasahara M., Takahashi Y., Nagata T. (2000). Thymidylate synthase expression correlates closely with *E2F1* expression in primary colon cancer. *Clinical Cancer Research*.

[30] Johnston P. G., Lenz H. J., Leichman C. G. (1995). Thymidylate synthase gene and protein expression correlate and are associated with response to 5-fluorouracil in human colorectal and gastric tumors. *Cancer Research*.

[31] Watanabe M., Sowa Y., Yogosawa M., Sakai T. (2013). Novel MEK inhibitor trametinib and other retinoblastoma gene (RB)-reactivating agents enhance efficacy of 5-fluorouracil on human colon cancer cells. *Cancer Science*.

