# Supplementary material for: CDK Inhibition Reverses Acquired 5-Fluorouracil Resistance in Hepatocellular Carcinoma Cells
Source: Dis Markers. 2022 Mar 11;2022:6907057. doi: 10.1155/2022/6907057 (PMC8933118; doi:10.1155/2022/6907057)
Supplement: Supplementary Materials — include CCK8 assay results for Figure 1 (BEL7402 vs. BEL7402/5-FU cells treated with 5-FU) and Figure 8 (BEL7402/5-FU cells treated with 5-FU vs. 5-FU-Dinaciclib). [file 6907057.f1.docx]

Annotation:

#Blank: empty plates

#control: plates with cells

#0.4uM - 1250uM: plates with cells treated with different concentration of 5-FU

#data in black: light absorbance values at 450 nm

#MEAN: the average of light absorbance values

#SD: standard deviation of light absorbance values

#inh%: inhibition rate calculated by (control - treatment)/(control - blank)

**Figure 1**

| **BEL-7402/5-FU cell line parallel test 1** | | | | | | | | |
| --- | --- | --- | --- | --- | --- | --- | --- | --- |
|  | Blank | Control | 39uM | 78uM | 156uM | 312.5uM | 625uM | 1250uM |
| 1 | 0.049 | 0.65 | 0.606 | 0.629 | 0.658 | 0.63 | 0.488 | 0.439 |
| 2 | 0.048 | 0.682 | 0.67 | 0.697 | 0.613 | 0.611 | 0.54 | 0.429 |
| 3 | 0.045 | 0.69 | 0.653 | 0.615 | 0.536 | 0.565 | 0.497 | 0.46 |
| 4 | 0.047 | 0.64 | 0.635 | 0.651 | 0.631 | 0.56 | 0.527 | 0.449 |
| MEAN | 0.05 | 0.67 | 0.64 | 0.65 | 0.61 | 0.59 | 0.51 | 0.44 |
| SD | 0.00 | 0.02 | 0.03 | 0.04 | 0.05 | 0.03 | 0.02 | 0.01 |
| **inh%** |  | **0.0%** | **4.0%** | **2.8%** | **9.1%** | **12.0%** | **24.7%** | **35.8%** |

| **BEL-7402/5-FU cell line parallel test 2** | | | | | | | | | |
| --- | --- | --- | --- | --- | --- | --- | --- | --- | --- |
|  | Blank | Control | 39uM | 78uM | 156uM | 312.5uM | 625uM | 1250uM | 2500uM |
| 1 | 0.044 | 0.6 | 0.636 | 0.597 | 0.65 | 0.582 | 0.52 | 0.456 | 0.334 |
| 2 | 0.046 | 0.539 | 0.589 | 0.575 | 0.621 | 0.555 | 0.481 | 0.442 | 0.31 |
| 3 | 0.045 | 0.521 | 0.606 | 0.58 | 0.586 | 0.538 | 0.489 | 0.42 | 0.298 |
| 4 | 0.047 | 0.513 | 0.57 | 0.571 | 0.556 | 0.531 | 0.494 | 0.454 | 0.287 |
| MEAN | 0.05 | 0.54 | 0.60 | 0.58 | 0.60 | 0.55 | 0.50 | 0.44 | 0.31 |
| SD | 0.00 | 0.04 | 0.03 | 0.01 | 0.04 | 0.02 | 0.02 | 0.02 | 0.02 |
| **inh%** |  | **0.0%** | **-11.5%** | **-7.5%** | **-12.1%** | **-1.7%** | **9.5%** | **20.1%** | **47.4%** |

| **BEL-7402 cell line parallel test 1** | | | | | | | | | | | | |
| --- | --- | --- | --- | --- | --- | --- | --- | --- | --- | --- | --- | --- |
|  | Blank | Control | 0.4uM | 0.8uM | 1.625uM | 3.125uM | 12.5uM | 25uM | 50uM | 100uM | 150uM | 200uM |
| 1 | 0.05 | 1.587 | 1.548 | 1.196 | 0.819 | 0.646 | 0.453 | 0.543 | 0.484 | 0.462 | 0.352 | 0.261 |
| 2 | 0.044 | 1.634 | 1.47 | 1.242 | 0.83 | 0.647 | 0.547 | 0.521 | 0.469 | 0.462 | 0.334 | 0.264 |
| 3 | 0.046 | 1.732 | 1.482 | 1.239 | 0.828 | 0.646 | 0.559 | 0.505 | 0.474 | 0.418 | 0.35 | 0.253 |
| 4 | 0.045 | 1.735 | 1.541 | 1.162 | 0.818 | 0.68 | 0.606 | 0.535 | 0.465 | 0.438 | 0.351 | 0.259 |
| MEAN | 0.05 | 1.67 | 1.51 | 1.21 | 0.82 | 0.65 | 0.54 | 0.53 | 0.47 | 0.45 | 0.35 | 0.26 |
| SD | 0.00 | 0.07 | 0.04 | 0.04 | 0.01 | 0.02 | 0.06 | 0.02 | 0.01 | 0.02 | 0.01 | 0.00 |
| **inh%** |  | **0.0%** | **9.9%** | **28.4%** | **52.2%** | **62.6%** | **69.6%** | **70.5%** | **73.8%** | **75.5%** | **81.5%** | **86.9%** |

| **BEL-7402 cell line parallel test 2** | | | | | | | | | | | | |
| --- | --- | --- | --- | --- | --- | --- | --- | --- | --- | --- | --- | --- |
|  | Blank | Control | 0.4uM | 0.8uM | 1.625uM | 3.125uM | 12.5uM | 25uM | 50uM | 100uM | 150uM | 200uM |
| 1 | 0.044 | 1.579 | 1.433 | 0.979 | 0.749 | 0.487 | 0.406 | 0.398 | 0.413 | 0.31 | 0.274 | 0.215 |
| 2 | 0.044 | 1.591 | 1.355 | 1.025 | 0.704 | 0.498 | 0.48 | 0.372 | 0.394 | 0.36 | 0.34 | 0.289 |
| 3 | 0.044 | 1.638 | 1.284 | 1.136 | 0.721 | 0.531 | 0.483 | 0.388 | 0.4 | 0.423 | 0.272 | 0.229 |
| 4 | 0.047 | 1.721 | 1.362 | 1.092 | 0.781 | 0.594 | 0.455 | 0.457 | 0.492 | 0.448 | 0.365 | 0.22 |
| MEAN | 0.04 | 1.63 | 1.36 | 1.06 | 0.74 | 0.53 | 0.46 | 0.40 | 0.42 | 0.39 | 0.31 | 0.24 |
| SD | 0.00 | 0.06 | 0.06 | 0.07 | 0.03 | 0.05 | 0.04 | 0.04 | 0.05 | 0.06 | 0.05 | 0.03 |
| **inh%** |  | **0.0%** | **17.2%** | **36.2%** | **56.3%** | **69.6%** | **74.1%** | **77.4%** | **76.1%** | **78.6%** | **83.1%** | **87.8%** |

| **BEL-7402 cell line parallel test 3** | | | | | | | | | | | | |
| --- | --- | --- | --- | --- | --- | --- | --- | --- | --- | --- | --- | --- |
|  | Blank | Control | 0.4uM | 0.8uM | 1.625uM | 3.125uM | 12.5uM | 25uM | 50uM | 100uM | 150uM | 200uM |
| 1 | 0.044 | 1.455 | 1.222 | 0.972 | 0.676 | 0.523 | 0.45 | 0.398 | 0.383 | 0.344 | 0.25 | 0.18 |
| 2 | 0.044 | 1.394 | 1.165 | 1.002 | 0.689 | 0.506 | 0.442 | 0.387 | 0.354 | 0.344 | 0.249 | 0.192 |
| 3 | 0.045 | 1.466 | 1.248 | 1.021 | 0.671 | 0.519 | 0.451 | 0.401 | 0.367 | 0.363 | 0.249 | 0.21 |
| 4 | 0.045 | 1.571 | 1.342 | 0.99 | 0.706 | 0.567 | 0.493 | 0.423 | 0.425 | 0.407 | 0.284 | 0.229 |
| MEAN | 0.04 | 1.47 | 1.24 | 1.00 | 0.69 | 0.53 | 0.46 | 0.40 | 0.38 | 0.36 | 0.26 | 0.20 |
| SD | 0.00 | 0.07 | 0.07 | 0.02 | 0.02 | 0.03 | 0.02 | 0.02 | 0.03 | 0.03 | 0.02 | 0.02 |
| **inh%** |  | **0.0%** | **15.9%** | **33.3%** | **55.1%** | **66.1%** | **71.0%** | **74.9%** | **76.3%** | **77.6%** | **85.0%** | **88.9%** |

**Figure 8**

| **BEL-7402/5-FU parallel test 1** | | | | | | | | |
| --- | --- | --- | --- | --- | --- | --- | --- | --- |
|  | Blank | control |  | 78uM | 156uM | 312.5uM | 625uM | 1250uM |
| 1 | 0.052 | 1.31 |  | 1.206 | 1.172 |  |  |  |
| 2 | 0.048 | 1.296 |  | 1.237 | 1.248 | 1.109 | 0.929 | 0.878 |
| 3 | 0.048 | 1.211 |  | 1.246 | 1.167 | 1.067 | 0.963 | 0.891 |
| 4 | 0.048 | 1.168 |  | 1.145 | 1.124 | 1.021 | 0.889 | 0.825 |
| MEAN | 0.05 | 1.25 |  | 1.21 | 1.18 | 1.07 | 0.93 | 0.86 |
| SD | 0.00 | 0.07 |  | 0.05 | 0.05 | 0.04 | 0.04 | 0.03 |
| **inh%** |  | **0.0%** |  | **3.2%** | **5.7%** | **15.1%** | **26.7%** | **31.9%** |

| **BEL-7402/5-FU parallel test 2** | | | | | | | | |
| --- | --- | --- | --- | --- | --- | --- | --- | --- |
|  | Blank | control |  | 78uM | 156uM | 312.5uM | 625uM | 1250uM |
| 1 | 0.049 | 1.167 |  | 1.111 | 1.024 |  |  |  |
| 2 | 0.047 | 1.223 |  | 1.179 | 0.977 | 0.94 | 0.869 | 0.804 |
| 3 | 0.047 | 1.211 |  | 1.12 | 0.977 | 0.994 | 0.807 | 0.788 |
| 4 | 0.047 |  |  | 1.186 | 0.91 | 1.07 | 0.889 | 0.706 |
| MEAN | 0.05 | 1.20 |  | 1.15 | 0.97 | 1.00 | 0.86 | 0.77 |
| SD | 0.00 | 0.03 |  | 0.04 | 0.05 | 0.07 | 0.04 | 0.05 |
| **inh%** |  | **0.0%** |  | **4.5%** | **19.8%** | **17.3%** | **30.0%** | **37.7%** |

| **BEL-7402/5-FU parallel test 3** | | | | | | | | |
| --- | --- | --- | --- | --- | --- | --- | --- | --- |
|  | Blank | control |  | 78uM | 156uM | 312.5uM | 625uM | 1250uM |
| 1 | 0.048 | 1.173 |  | 1.342 | 1.164 | 1.242 | 1.041 | 0.799 |
| 2 | 0.047 | 1.322 |  | 1.186 | 1.19 | 1.102 | 0.929 | 0.865 |
| 3 | 0.047 | 1.181 |  | 1.199 | 1.042 | 1.228 | 0.955 | 0.877 |
| 4 | 0.047 |  |  |  | 1.006 | 1.087 | 1.07 | 0.826 |
| MEAN | 0.05 | 1.23 |  | 1.24 | 1.10 | 1.16 | 1.00 | 0.84 |
| SD | 0.00 | 0.08 |  | 0.09 | 0.09 | 0.08 | 0.07 | 0.04 |
| **inh%** |  | **0.0%** |  | **-1.4%** | **10.6%** | **5.1%** | **19.2%** | **32.6%** |

| **BEL-7402/5-FU + Dinaciclib 10nmol/L parallel test 1** | | | | | | | |
| --- | --- | --- | --- | --- | --- | --- | --- |
|  | Blank | control | Dinaciclib 10nm | Dinaciclib 10nm+5-FU 78uM | 156uM | 312.5uM | 625uM |
| 1 | 0.052 | 1.31 | 1.229 | 1.078 | 0.981 | 0.931 | 0.849 |
| 2 | 0.048 | 1.296 | 1.24 | 1.198 | 1.139 | 1.002 | 0.852 |
| 3 | 0.048 | 1.211 | 1.34 | 1.22 | 1.099 | 0.992 | 0.87 |
| 4 | 0.048 | 1.168 |  |  | 0.939 | 1.073 | 0.863 |
| MEAN | 0.05 | 1.25 | 1.27 | 1.17 | 1.04 | 1.00 | 0.86 |
| SD | 0.00 | 0.07 | 0.06 | 0.08 | 0.09 | 0.06 | 0.01 |
| **inh%** |  | **0.0%** | **-2.0%** | **6.8%** | **17.3%** | **20.6%** | **32.4%** |

| **BEL-7402/5-FU + Dinaciclib 10nmol/L parallel test 2** | | | | | | | | |
| --- | --- | --- | --- | --- | --- | --- | --- | --- |
|  | Blank | control | Dinaciclib 10nm | Dinaciclib+5-FU 78uM | 156uM | 312.5uM | 625uM | 1250uM |
| 1 | 0.049 | 1.167 | 1.126 | 1.096 | 0.822 | 0.765 | 0.789 | 0.661 |
| 2 | 0.047 | 1.223 | 1.127 | 1.026 | 1.135 | 0.91 | 0.793 | 0.774 |
| 3 | 0.047 | 1.211 | 1.26 | 1.084 | 0.922 | 0.942 | 0.764 | 0.686 |
| 4 | 0.047 |  |  |  | 0.796 | 0.717 | 0.765 | 0.749 |
| MEAN | 0.05 | 1.20 | 1.17 | 1.07 | 0.92 | 0.83 | 0.78 | 0.72 |
| SD | 0.00 | 0.03 | 0.08 | 0.04 | 0.15 | 0.11 | 0.02 | 0.05 |
| **inh%** |  | **0.0%** | **2.5%** | **11.4%** | **24.4%** | **31.8%** | **36.7%** | **41.9%** |

| **BEL-7402/5-FU + Dinaciclib 10nmol/L parallel test 3** | | | | | | | | |
| --- | --- | --- | --- | --- | --- | --- | --- | --- |
|  | Blank | control | Dinaciclib 10nm | Dinaciclib+5-FU 78uM | 156uM | 312.5uM | 625uM | 1250uM |
| 1 | 0.048 | 1.173 | 1.273 | 1.266 | 1.065 | 1.121 | 0.924 | 0.778 |
| 2 | 0.047 | 1.322 | 1.069 | 1.01 | 1.027 | 1.039 | 0.876 | 0.805 |
| 3 | 0.047 | 1.181 | 1.094 | 1.241 | 1.027 | 0.891 | 0.832 | 0.704 |
| 4 | 0.047 |  |  |  |  | 0.873 | 0.759 | 0.673 |
| MEAN | 0.05 | 1.23 | 1.15 | 1.17 | 1.04 | 0.98 | 0.85 | 0.74 |
| SD | 0.00 | 0.08 | 0.11 | 0.14 | 0.02 | 0.12 | 0.07 | 0.06 |
| **inh%** |  | **0.0%** | **6.8%** | **4.5%** | **15.8%** | **20.7%** | **32.1%** | **41.2%** |
